# ACCEPTABILITY DATA OF A TECHNOLOGY-ENABLED CARE COACHING SERVICE FOR CAREGIVERS OF INDIVIDUALS WITH DEMENTIA

**DOI:** 10.1093/geroni/igac059.3105

**Published:** 2022-12-20

**Authors:** Katherine Judge, Amelia Thomley, Lauren Stratton, Emily Silver, Kerry Finegan, Monica Moreno, Sam Fazio, Joe Chung

**Affiliations:** Cleveland State University, Cleveland, Ohio, United States; Kinto, Cambridge, Massachusetts, United States; Alzheimer’s Association, Chicago, Illinois, United States; Kinto, Cambridge, Massachusetts, United States; Alzheimer’s Association, Chicago, Illinois, United States; Alzheimer’s Association, Chicago, Illinois, United States; Alzheimer’s Association, Chicago, Illinois, United States; Kinto, Cambridge, Massachusetts, United States

## Abstract

This poster presents acceptability data from the Kinto Care Coaching Intervention, an innovative technology-enabled care coaching service for caregivers of individuals with dementia. The 30-day program provides caregiver support and financial information through: 1) an initial one-on-one care coaching meeting; 2) interactive and on-going support and educational resources through an app; 3) access to support groups; and 4) if needed, additional meetings with their care coach. The solution is funded through the NIH’s Small Business Innovation and Research (SBIR) program, with the specific goals of developing mobile technology that is: 1) acceptable and usable for caregivers of all ages and 2) supports cost-effective deployment of the coaching intervention at scale. To assess program acceptability, participants completed a survey after their one-on-one care coaching meeting and after the 30-day program. On average, participants (n=32) were M=51.94 (SD=12.07) years old; 68.8% female; 71.9% White; 75% married; and 56.3% worked full-time. Using a 5-point Likert scale, nearly all participants (96.6%) indicated they ‘agreed’ or ‘strongly agreed’ that their care coach was: helpful in explaining the program; provided useful information; assisted with developing goals; and were supportive. Participants rated the program resources and technology as very helpful with mean ratings ranging from 4.41 to 4.69. When asked about the overall program acceptability, participants indicated they were extremely satisfied with the program (M=4.81; SD=.40), with 100% of participants ‘agreed’ or ‘strongly agreed’ that they were satisfied. Discussion will highlight key program components along with next steps in testing program efficacy.

